# Belladonna in Preventing the Secretion of Milk, and in Mammary Abscess

**Published:** 1859-12

**Authors:** A. Hard


### ECLECTIC DEPARTMENT,

### AND SPIRIT OF THE MEDICAL PERIODICAL PRESS.

---

*Belladonna in preventing the secretion of Milk, and in Mammary Abscess.* By A. Hard, M.D. (Read before the Aurora City Medical Association, at Aurora, Ill., Oct. 3, 1859.)

There has been much said of late of the virtues of belladonna in preventing and arresting the secretion of milk; and having, with others, labored under the inconvenience of possessing no reliable remedy to fulfill such indications, I resolved to test its virtues; and I propose to give the result of its use in six cases, in which I have endeavored to give it a fair trial.

In the first three it was desirable to prevent the secretion of milk in one breast (from the entire want of development of the nipple), without interfering with the other. All three patients had previously suffered from mammary abscesses, and they looked forward to their approaching accouchment with "fear and trembling." Immediately after delivery I ordered the following:

℞ Ext. belladonna, - - - ℈ ij.

Aqua font., - - - - ℥ ij.

Misce.

With this solution bathe the *one* breast every four hours, until the flow of milk is established in the other; then apply less frequently, but continue its use a week longer.

In one patient, there was no milk secreted in the breast thus treated, while the other breast afforded the usual amount, and appeared unaffected. In the other two cases there was a little milk secreted, but not sufficient to distend the gland so as to produce inconvenience; while the opposite breasts, like that of the first case, performed their functions unimpaired. The fourth was a case of premature delivery at the sixth month, as the result of placenta prævia. The solution of belladonna was applied to both breasts. But little milk was secreted, which was soon absorbed.

The fifth was a case of abortion. The belladonna was not applied until the breasts became distended and painful. I ordered that the milk be drawn with a breast-pump once; then applied the belladonna, and had no further trouble.

In the last case, a large abscess had formed in one breast, which was discharging, and extensive swelling and inflammation existed in the other, at the time I was called. The patient had been a great sufferer for four weeks, both from the "cold water treatment" and the mammary inflammation. I applied the belladonna as in the other cases; also, warm fomentations; had the bowels moved by sulph. magnesia, and ordered the following:

℞ Sulph. quinine, - - grs. 15.
Pulv. opii, - - - - " 2.
Misce.

Div. in chart. No. 4. Dose, one in every three hours.

Under this treatment suppuration was prevented, the inflammation was subdued, and convalescence soon followed. There was but little constitutional effect of belladonna perceptible. In two cases, the pupils of the eyes were a little dilated; the children were not affected by it. However, I took the precaution to guard against its inhalation either by the mother or child.

If belladonna proves as useful in the hands of others as it has in mine, in these unpleasant complications consequent upon child-bearing, a vast amount of pain and suffering will be mitigated, if not entirely prevented. I would call the attention of the Association particularly, to its application to one breast, leaving the other undisturbed.

